# Occurrence of NDM-1 and VIM-2 Co-Producing *Escherichia coli* and OprD Alteration in *Pseudomonas aeruginosa* Isolated from Hospital Environment Samples in Northwestern Tunisia

**DOI:** 10.3390/diagnostics11091617

**Published:** 2021-09-04

**Authors:** Raouaa Maaroufi, Olfa Dziri, Linda Hadjadj, Seydina M. Diene, Jean-Marc Rolain, Chedly Chouchani

**Affiliations:** 1 MEPHI, IHU Méditerranée Infection, 19-21, Boulevard Jean Moulin, Aix-Marseille Université, 13005 Marseille, France; maaroufiraoua@hotmail.com (R.M.); linda.hadjadj@univ-amu.fr (L.H.); seydina.diene@univ-amu.fr (S.M.D.); jean-marc.rolain@univ-amu.fr (J.-M.R.); 2 Laboratoire des Microorganismes et Biomolécules Actives, Faculté des Sciences de Tunis, Campus Universitaire, Université de Tunis El-Manar, Tunis 2098, Tunisia; dziri_olfa@hotmail.fr; 3 Laboratoire de Recherche Sciences et Technologies de l’Environnement, Institut Supérieur des Sciences et Technologies de l’Environnement de Borj-Cedria, Technopôle de Borj-Cedria, Université de Carthage, PB 1003, Hammam-Lif 2050, Tunisia; 4 Unité de Service en Commun pour la Recherche «Plateforme génomique» Institut Supérieur des Sciences et Technologies de l’Environnement de Borj-Cedria, Technopôle de Borj-Cedria, Université de Carthage, PB 1003, Hammam-Lif 2050, Tunisia

**Keywords:** hospital environment, *Enterobacteriaceae*, NDM-1, VIM-2, OprD, Tunisia

## Abstract

Hospital environments constitute the main reservoir of multidrug-resistant bacteria. In this study we aimed to investigate the presence of Gram-negative bacteria in one Northwestern Tunisian hospital environment, and characterize the genes involved in bacterial resistance. A total of 152 environmental isolates were collected from various surfaces and isolated using MacConkey medium supplemented with cefotaxime or imipenem, with 81 fermenter bacteria (27 *Escherichia coli,* and 54 *Enterobacter* spp., including 46 *Enterobacter cloacae*), and 71 non-fermenting bacteria (69 *Pseudomonas* spp., including 54 *Pseudomonas aeruginosa,* and 2 *Stenotrophomonas maltophilia*) being identified by the MALDI-TOF-MS method. Antibiotic susceptibility testing was performed by disk diffusion method and E-Test was used to determine MICs for imipenem. Several genes implicated in beta-lactams resistance were characterized by PCR and sequencing. Carbapenem resistance was detected among 12 isolates; nine *E. coli* (*bla*_NDM-1_ (*n* = 8); *bla*_NDM-1_ + *bla*_VIM-2_ (*n* = 1)) and three *P. aeruginosa* were carbapenem-resistant by loss of OprD porin. The whole-genome sequencing of *P. aeruginosa* 97H was determined using Illumina MiSeq sequencer, typed ST285, and harbored *bla*_OXA-494_. Other genes were also detected, notably *bla*_TEM_ (*n* = 23), *bla*_CTX-M-1_ (*n* = 10) and *bla*_CTX-M-9_ (*n* = 6). These new epidemiological data imposed new surveillance strategies and strict hygiene rules to decrease the spread of multidrug-resistant bacteria in this area.

## 1. Introduction

Hospital environments are considered as the main reservoir of multidrug-resistant bacteria and play a major role in the acquisition of healthcare-associated infections, also known as nosocomial infections or hospital-acquired infections [1]. Nosocomial infections constitute a major health problem worldwide since they have been associated with a high rate of morbidity and mortality [2]. The incidence of nosocomial infections varies from 3.6 to 12% and 5.7 to 19.1% in high-income countries and in low- and middle-income countries, respectively [3]. Ninety percent of these infections are caused by bacteria [4]. Several studies have reported that Gram-negative bacteria constitute the principal cause of nosocomial infections, including urinary tract infections, pneumonia, bloodstream infections, and surgical infections, especially in the intensive care unit (ICU) [5]. Unfortunately, these infections are caused by multidrug-resistant Gram-negative bacteria, including carbapenemase and extended-spectrum β-lactamase (ESBL) producers, thus restricting the therapeutic options [6].

Interestingly, several studies have reported the detection of multidrug-resistant Gram-negative bacteria isolated from hospital environments in different regions across the world. These isolates belong to different genera of *Enterobacteriaceae*, *Pseudomonas* and *Acinetobacter*, and harbor various genes coding for ESBLs and carbapenemases, including metallo-β-lactamases [7]. Moreover, these genes are usually carried on plasmids and other mobile genetic elements, such as transposons and integrons, which facilitates their rapid spread among different Gram-negative species [8]. In Tunisia, there are few studies reporting the dissemination of carbapenemase and ESBL encoding genes among multidrug-resistant Gram-negative bacteria isolated from hospital environments. Thus, these strains were mainly imipenemase (IMP) producing *Klebsiella pneumoniae*, the Verona integron-encoded metallo-beta-lactamase (VIM-2) producing *E. coli* [9], and the cefotaximase (CTX-M-15) producing *Enterobacteriaceae* [10]. Nevertheless, epidemiological studies focusing on the hospital environment are absent in several regional hospitals, especially in northwestern Tunisia. As previously described, these bacteria are innately able to survive in the hospital environment, including hand-touch sites and medical devices, and are not totally eradicated even by cleaning with bleach [1]. This reflects the importance of routine screening and monitoring of high-risk surfaces to generate targeted cleaning in order to prevent the transmission of multidrug-resistant bacteria to healthy people as well as to patients and especially hospitalized patients [1]. These previous studies exhorted us to investigate the prevalence of Gram-negative bacteria in the hospital environment in the Jendouba governorate, located in northwestern Tunisia, and characterize genes involved in β-lactam resistance, including carbapenems, constituting the main goal of our study. To our knowledge, this is the first epidemiological study conducted in this region, which is characterized by a 135 km Algerian border zone in the west and a 25 km Mediterranean coastline in the north.

## 2. Material and Methods

### 2.1. Swabs Sampling and Bacterial Isolation

Between February and December of 2017, a total of 162 swabs were sampled from environmental surfaces and equipment in different wards of the regional hospital of Jendouba in northwestern Tunisia. These samples were collected from the surgery ward (orthopedic, visceral, gynecology, and revealing room), the intensive care unit (3 rooms) and the gastroenterology ward (rectoscopy and fibroscopy).

The sampling was performed using sterile cotton swabs moistened with sterile saline. These swabs were stored at 4 °C and transported directly to the University laboratory in order to achieve the isolation step. Collected samples were incubated in 10 mL of brain heart infusion broth medium (BioMérieux, Marcy l’Etoile, France) for 48 h at 37 °C. The broth samples were then cultured on MacConkey agar medium (BioMérieux, Marcy l’Etoile, France), supplemented with cefotaxime (2 mg/L) and imipenem (2 mg/L) (Bio-Rad, Marnes-la-Coquette, France), in order to isolate third-generation cephalosporin (3GC) and carbapenem-resistant Gram-negative bacteria, respectively. Following the incubation for 24 h at 37 °C, we obtained morphologically different colonies (differing by color, size, shape) in each plate from only 78 swabs. Well isolated colonies, growing on the selective media, were taken separately and purified. This step allowed us to obtain a total of 152 isolates that were identified using the matrix-assisted laser desorption/ionization time-of-flight mass spectrometry (MALDI-TOF-MS) method (MicroflexTM; Bruker Daltonics, Bremen, Germany), with the flexcontrol and the biotyper 3.0 software (Bruker Daltonics).

### 2.2. Antibiotic Susceptibility Testing

Antibiotic susceptibility testing was carried out on Mueller–Hinton agar (BioMérieux, Marcy l’Etoile, France) using the standard disk diffusion method, according to the Antibiogram Committee of the French Society for Microbiology (EUCAST/CA-SFM 2017) (http://www.sfm-microbiologie.org/) and the Clinical and Laboratory Standards Institute (CLSI 2017) guidelines (https://www.clsi.org/). Two different panels of 16 antibiotic disks (Bio-Rad, Marnes-la-Coquette, France) were used for fermenter GNB and non-fermenting GNB, respectively, as follows: Amoxicillin (AMX), Amoxicillin-Clavulanic Acid (AMC), Piperacillin + tazobactam (TZP), Cephalothin (KF), Ceftriaxone(CRO), Pefloxacin (PEF), Ertapenem (ETP), Imipenem (IPM), Colistin (CS), Amikacin (AK), Gentamicin(CN), Ciprofloxacin (CIP), Fosfomycin (FF), Nitrofurantoin (F. NIT), Doxycycline (DO), Trimethoprim-Sulfamids (SXT); and Ticarcillin (TIC), Ticarcillin + Clavulanic Acid (TIM), Piperacillin + tazobactam (TZP), Ceftazidime (CAZ), Pefloxacin (PEF), Imipenem (IPM), Colistin (CS), Amikacin (AK), Tobramycin (TOB), Gentamicin (CN), Ciprofloxacin (CIP), Fosfomycin (FF), Nitrofurantoin (F. NIT), Doxycycline (DO), Trimethoprim-Sulfamids (SXT), Rifampicin (RA).

Moreover, the double-disk synergy test (DDST) and CarbaNP were used to investigate ESBL and carbapenemase production, respectively. Furthermore, minimum inhibitory concentrations (MICs) were performed by ETEST^®^ MBL strip (BioMérieux, Marcy l‘Etoile, France) method for carbapenem-resistant isolates screening. MICs results were interpreted, using the CLSI breakpoints. Results of colistin susceptibility were validated using MIC by the broth microdilution according to the joint CLSI-EUCAST Polymyxin Breakpoints Working Group and we used two recommended quality control strains: *E. coli* ATCC 25922, and *E. coli* NCTC 13846 (mcr-1 positive). Additionally, we used a Beta CARBA test (the β-CARBA™ test; Bio-Rad) for rapid detection of carbapenemase. It is based on the change of color of a chromogenic substrate in presence of CPE. The test was performed in a micro-tube from the three freshly isolated carbapenem-resistant *P. aeruginosa* isolates colonies. The interpretation was performed at 30 min, after 1 h, and after 24 h of incubation at 37 °C.

### 2.3. Molecular Characterization of Resistance Genes

The molecular characterization of carbapenemase encoding genes (*bla*_KPC_, *bla*_IMP_, *bla*_VIM_, *bla*_NDM_, *bla*_OXA-48_, *bla*_OXA-23,_ *bla*_OXA-24_ and *bla*_OXA-58)_ and for the OprD gene was performed by conventional polymerase chain reaction (PCR) using specific primers as described previously [11,12], followed by sequencing based on the Sanger method. All bacteria were also screened for β-lactamases (*bla*_CTX-M_, *bla*_TEM_, and *bla*_SHV_) genes and for *mcr*-1, 2, 3, 4 and 5 genes [13,14]. The amplicons of each tested gene were sequenced using BigDye1 terminator chemistry on an automated ABI 3130 sequencer (PE Applied Biosystems, Foster City, CA, USA). Sequences were compared with those available in the database (GenBank). The sequences were then aligned using the npsa_clustalW, the Network Protein Sequence Analysis (https://npsa-prabi.ibcp.fr/), to analyze the amino acid sequences translated into proteins. The PROVEAN software (http://provean.jcvi.org/index.php) was used to check whether amino acid sequence changes could induce an alteration of protein function.

### 2.4. Whole-Genome Sequencing and Genomic Comparison

Genomic DNA was extracted by EZ1 Extraction using the Kit EZ1^®^ DNA (Qiagen, Courtaboeuf, France). Whole-genome sequencing (WGS) was performed by Illumina MiSeq sequencer (Illumina, San Diego, CA, USA) for one carbapenem-resistant *P. aeruginosa* isolate (97H ST285), which was found OXA-48 encoding gene positive by PCR. This WGS was assembled by A5 assembler. The PROKKA software was used for the annotation, the ARG-ANNOT program was used to identify antibiotic resistance genes, and the Plasmid Finder program was used to detect the presence of plasmids in the genome of the isolate. For phylogenetic analysis, the conserved proteins of carbapenemase encoding genes in relation to those of many bacterial families, downloaded from the NCBI assembly database (BlastP), were selected and independently aligned using MUSCLE to cluster homologous genes.

## 3. Results

### 3.1. Bacterial Isolates

Over the study period, which extended from February to December of 2017, and among the total of 162 sampled swabs, only 78 swabs were positive for the presence of Gram-negative bacteria (48.15%), detecting a total of 152 isolates. These samples were obtained after routine cleaning from various surfaces which were in direct contact with patients and all sanitary staff, from three different wards (the surgery ward, the intensive care unit, and the gastroenterology ward) as described in Table 1. The bacterial isolates were detected on many surfaces, and they were most frequently obtained from oxygen masks (*n* = 18), followed by serum supports (*n* = 16), and material trays (*n* = 11), as shown in Table 1. The identification of these isolates by MALDI-TOF showed the detection of 81 fermenter GNB distributed as follows: 27 *E. coli,* 54 *Enterobacter* spp. including 46 *E. cloacae,* as well as 71 non-fermenting GNB, mainly 69 *Pseudomonas* spp. including 54 *P. aeruginosa,* and 2 *S. maltophilia.*

### 3.2. Phenotypic Characterization of Antibiotic Resistance

All environmental GNB isolates showed resistance to many antibiotic families as described in Table 2 and Table 3. They showed differences in antibiotic resistance phenotype. Antibiotic susceptibility testing results revealed high and different levels of resistance rates of fermentative GNB against tested antibiotics, such as amoxicillin, amoxicillin-clavulanic acid, cephalothin, pefloxacin, gentamicin, ciprofloxacin, and trimethoprim-sulfamid (Table 2). In addition, the non-fermenting GNB isolates showed resistance to beta-lactams and other antibiotics families, but not with the same level of fermenter GNB resistance (Table 3). Twelve carbapenem-resistant Gram-negative environmental isolates, identified as *E. coli* (*n* = 9) and *P. aeruginosa* (*n* = 3), were detected. Most of them were isolated from the reanimation rooms. These rooms should be sterile to avoid nosocomial infections. The presence of Gram-negative bacteria, especially multidrug-resistant isolates, in these reanimation rooms after routine cleaning from various surfaces which were in direct contact with patients and all sanitary staff, constitutes a real concern. E-tests showed a high level of resistance to imipenem, with MIC > 32 μg/mL for all isolates. None of the isolates were resistant to colistin (MIC < 2 μg/mL). With regard to the rapid Beta CARBA test for the three carbapenem-resistant *P. aeruginosa* isolates, there was a variation observed in interpretation of MBL-producers results after different incubation times. Positive results were obtained after 30 min of incubation.

It is also very interesting to note the presence of several isolates that are classified as intermediate or even sensitive in this hospital environment (Table 2 and Table 3). These isolates should not be ignored because they could cause nosocomial infections and acquire resistance genes under selective pressure, by mutations, or even horizontal transfer via mobile genetic elements, owing to the plasticity of the bacterial genome.

### 3.3. Molecular Characterization of Resistance Genes

Multidrug-resistant bacteria, especially carbapenemase and ESBL producers, were mainly detected in the ICU, gastroenterology and surgery wards. As described in Table 4, these isolates were obtained from different surfaces, including oxygen masks and serum supports. The molecular characterization of carbapenemase encoding genes by PCR, showed that the NDM-1 encoding gene was detected in 9 *E. coli*, and one of these isolates co-harbored the metallo-beta-lactamase encoding gene *bla*_VIM-2_ (Table 4).

Other ESBL encoding genes were also detected. Thus, eleven *E. coli* harbored CTX-M encoding genes, the *bla*_CTX-M-9-like_ (*n* = 6) and *bla*_CTX-M-1-like_ (*n* = 5) genes. CTX-M-1-like enzyme was also detected among five *E. cloacae*. Moreover, TEM was the most common detected beta-lactamase (*n* = 23), distributed as follows; 16 *E. coli*, 2 *P. aeruginosa*, 1 *P. stutzeri,* and 4 *E. cloacae*. We detected 6 TEM variants including *bla*_TEM-1_ (*n* = 2), *bla*_TEM-163_ (*n* = 8), *bla*_TEM-104_ (*n* = 3), *bla*_TEM-198_ (*n* = 7), *bla*_TEM-164_ (*n* = 2), and *bla*_TEM-116_ (*n* = 1). However, all isolates were negative for KPC, CTX-group B, SHV, OXA-24, and OXA-23 enzymes. They were also negative for *mcr*-genes.

**Figure 1:**
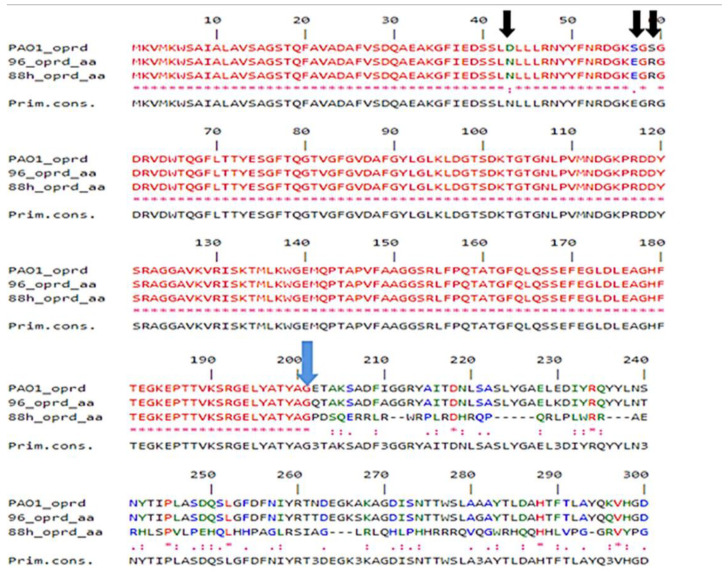
Protein Comparison by NPSA-CLUSTALW between our sequences of OprD gene with wild-type porin sequence from strain PAO1.

OprD gene mutations were detected in the two carbapenem-resistant *P. aeruginosa* isolates, 96H and 88H. The obtained OprD gene sequences were compared with the wild-type OprD gene of PAO1 isolate. Thus, the comparison of OprD protein sequences using the Network Protein Sequence Analysis NPSA-CLUSTALW (https://npsa-prabi.ibcp.fr/) revealed that chromosomal mutations were installed, conducting to the neutral base substitutions in the porin structure (D43N, S57E, and S59R), indicated by three black arrows, and a creation of a stop codon in the position 201-pb, which is designated by a blue arrow (Figure 1).

### 3.4. Whole-Genome Sequence Analysis and Genomic Comparison

With regard to the carbapenem-resistant *P. aeruginosa* isolates 97H ST285, the standard PCR targeting carbapenem resistance encoding genes was positive for not only OprD porin but also for *bla*_OXA-48_. To prove this important result obtained by PCR, antibiotic susceptibility testing was repeated. It revealed that this isolate was resistant to imipenem (IPM) (MIC/IPM > 32μg/mL) and ertapenem (ETP) (MIC/ETP > 32μg/mL). It remained susceptible to ticarcillin-clavulanic acid, piperacillin-tazobactam, pefloxacin, ceftazidime, ceftriaxone, and meropenem, as described by the Antibiogram Committee of the French Society for Microbiology (CA-SFM 2017). PCR products sequencing and BLASTn analysis revealed that this isolate harbored a *bla*_OXA-48_-like gene with 94.12% identity (AQA26426.1).

**Figure 2:**
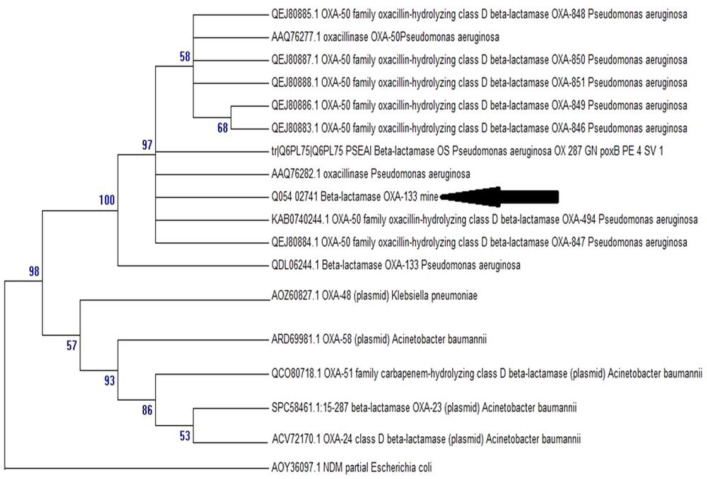
Phylogenetic tree including our sequence, variants of OXA-50 and representatives of the main groups of oxacillinases.

The whole-genome sequencing (WGS) was assembled into 65 scaffolds and 65 contigs leading to a genome size of 6′224′225-bp and GC content of 66.5%. Genomic analysis revealed that this isolate belonged to the ST285 clone. This isolate did not contain any plasmid. We detected by ARG-ANNOT program that this pathogen exhibit 100% nucleotide identity with the published OXA-50 family oxacillin-hydrolyzing class D beta-lactamase OXA-494 from *P. aeruginosa* (WP_003118452.1). The variant sequence *bla*_OXA-494_ gene differs from OXA-50 by two amino acids (D109E and R167H). In addition, to investigate the phylogenetic relationship of this *bla*_OXA-494_ variant from the 97H isolate, a phylogenetic tree with proteins belonging to the variants of OXA-50 and representatives of the main groups of oxacillinases families was reconstructed. As shown in Figure 2 of the phylogenetic tree, our identified and annotated sequence and OXA-50 variants, grouped together with proteins as the OXA-50 family, where thus demonstrating their membership. Additionally, the analysis of the obtained sequence of the OprD encoding gene, as well as the corresponding protein sequence among this *P. aeruginosa* 97H isolate, showed the installation of amino acids substitutions in seventeen different positions (S57E, S59R, I210A, E230K, S240T, N262T, A267S, A281G, K296Q, R310G, V359L, N375S, N376S, V377S, G378S, Y382A, and G383C) when compared to the wild-type porin sequence from strain PAO1.

Moreover, the genome comparison with the available sequences of *P. aeruginosa* in the NCBI database showed that this pathogen exhibits other antibiotic resistance genes which were identified as: for beta-lactams *bla*_TEM-116_, for aminoglycoside *agly*_aph3-iib_ and for phenicol *phe*_catb7_, respectively, with percentage identities 100.000%, 97.89% and 98.91%. To date, this study presents the first report published of this result in Tunisia.

## 4. Discussion

Gram-negative bacteria, including *Enterobacteriaceae*, are able to survive on abiotic surfaces which present a pathogenic transmitter from one surface to another, or even from the environmental area to patients and to staff [1]. This has been confirmed by several studies, as well as our findings, which reveal a high incidence of *Enterobacteriaceae* in 50% of environmental samples, taking into account all the swabs collected. Moreover, we have revealed the predominance of *P. aeruginosa* (35.5%), followed by *E. cloacae* (30.2%), *E. coli* (17.8%), *Pseudomonas* spp. (9.9%), *Enterobacter* spp. (5.3%), and *S. maltophilia* (1.3%) on different surfaces of this northwestern hospital, noting the absence of *K. pneumoniae* species. Previous studies have also shown the detection of these species causing infections in hospitals in Tunisia and in other countries in the world [1,10]. These isolates were mainly collected from oxygen masks, followed by serum supports and material trays. What is more, we have reported in the present study the detection of different environmental isolates harboring carbapenemase encoding genes and 3GC. The spread of multidrug-resistant Gram-negative bacteria, especially carbapenemases and ESBL producers, in the hospital environment has been recorded in different countries, including Tunisia [7,9,10,15].

In this study, only twelve carbapenem-resistant isolates were detected and identified as *E. coli* and *P. aeruginosa*. All our carbapenem-resistant *E. coli* isolates were NDM-1 producers, one of these isolates co-produced VIM-2. To our knowledge, NDM-1 producing *E. coli* has not been reported hitherto in Tunisia. This enzyme was mainly detected among *K. pneumoniae* and *A. baumannii* clinical isolates [16]. This is the first study reporting the co-production of these two metallo-β-lactamases, NDM-1 and VIM-2, in our country. However, a recent Tunisian study described the detection of NDM-1 and VIM-1 co-producing *K. pneumoniae* clinical isolates [17]. In addition, previous Tunisian data showed that several multidrug-resistant Gram-negative isolates were detected in environmental samples from Tunisian hospitals, and they were mainly identified as IMP producing *K. pneumoniae*, VIM-2 producing *E. coli* [9], and CTX-M-15 producing *Enterobacteriaceae* [10]. Since 2009, the date of the first description of NDM-1 in Indian *E. coli* and *K. pneumoniae* clinical isolates [18], this enzyme has been dramatically disseminated worldwide among various species of Gram-negative bacteria [19,20,21]. The rapid dissemination of these genes within *Enterobacteriaceae* and different species of Gram-negative bacteria is due to their association to the mobile genetic elements, especially plasmids, facilitating their transmission [22,23]. It is also very interesting to note the detection of these genes in the hospital effluents [24].

The loss of OprD porin among our *P. aeruginosa* isolates constitutes the main mechanism conferring resistance to carbapenems. As shown by several studies, the deficiency of OprD production was mainly the result of mutations or insertion sequences disrupting the *OprD* gene or even its promoter region [25,26,27]. Compared with wild-type porin from strain PAO1, neutral base substitutions were found exhibiting a reduced amount of OprD in the outer membrane and increased carbapenem resistance, as previously described [28,29]. *P. aeruginosa* is considered an important opportunistic pathogen and one of the major causes of community and nosocomial infections with limited therapeutic options [30,31]. This pathogen employs several beta-lactam resistance mechanisms, including multi-drug efflux pump overexpression, restricted membrane permeability, alteration in the penicillin-binding proteins, and production of beta-lactamases [32].

Treatment of infections caused by such a pathogen often proves challenging and seems to be ineffective, as this micro-organism exhibits an innate and acquired resistance to a broad range of antibiotics. With the advent of whole-genome sequencing, a chromosomally encoded oxacillinase, the OXA-50 enzyme type, was documented in *P. aeruginosa* [33]. The contribution of this enzyme in beta-lactam resistance has not been completely elucidated. The gene coding for this enzyme is constitutively expressed for the first time in the world in *P. aeruginosa* [34]. It was also shown that this gene confers decreased susceptibility to ampicillin and ticarcillin, as well as to moxalactam and meropenem only in *P. aeruginosa* but not in *E. coli* [34]. Among the OXA-50 variants, there were two enzymes, OXA-494 and OXA-488, which showed an activity against imipenem but not meropenem, ticarcillin and third-generation cephalosporins [30,34]. Therefore, we report in the present study for the first time in Tunisia the *bla*_OXA-50_ and *bla*_OXA-494_ genes in *P. aeruginosa.* *bla*_OXA-50 h_ allele from the ST285 isolate differed by five and four nucleotides, respectively, from the *bla*_OXA-50_ gene of *P. aeruginosa* PAO1 [34], and their closest relative was poxB27, previously identified in the *P. aeruginosa* strain DSM 1253 of unreported origin [35]. The molecular characterization of *bla*_OXA-50_ gene could be another potential clonality marker for *P. aeruginosa* [30]. To our knowledge, the *bla*_OXA-50_ gene has not been hitherto reported in Tunisia.

The present study reveals the spread of ESBL producing Gram-negative bacteria especially in *E. coli* isolates [36]. Similarly, many studies described that this has increased significantly in different countries worldwide [7].

In our study, TEM-type beta-lactamase was the most common detected enzyme among various species including *E. coli*, *E. cloacae*, *P. aeruginosa*, and *P. stutzeri*. Thus, we have detected six TEM variants in our isolates, but only two were qualified as ESBLs: TEM-164 and TEM-116. These ESBL variants were previously found in *K. pneumoniae* and *Providencia stuartii* clinical isolates, respectively, in Tunisia [37,38]. However, this study reports for the first time the detection of *bla*_TEM-116_ among *P. aeruginosa* and *bla*_TEM-164_ among *E. coli* in our country.

Similarly, two CTX-M-type ESBLs were also detected in our study, with the CTX-M-1-like enzyme being identified among *E. coli* and *E. cloacae*. However, the CTX-M-9-like enzyme was detected only among *E. coli*. Several studies have shown the high prevalence of the CTX-M producing *Enterobacteriaceae* isolated from patients or even from the hospital environment, noting the dominance of CTX-M-15 [39,40].

## 5. Conclusions

Overall, the present study reveals the dissemination of multidrug-resistant Gram-negative bacteria in the hospital environment, including carbapenemases and ESBL producers. Our research also provides new epidemiological data showing the quick spread of several genes among bacterial isolates, noting *bla*_NDM-1_, *bla*_VIM-2_, *bla*_OXA-494,_ *bla*_TEM_, *bla*_CTX-M-1-like_ and *bla*_CTX-M-9-like_ in this region. The detection of multidrug-resistant bacteria in the ICU and in the gastroenterology and surgery wards constitutes a real threat that must be taken into consideration and priority should be given for interventions to reduce the level of nosocomial infections. It is also very interesting to note the presence of this large number of resistant bacteria harboring various resistance genes in the environment of a small hospital, where the patient accommodation capacity is less than that of the capital hospitals.

These findings reveal that this regional hospital could be a reservoir of multidrug-resistant bacteria as well as a potential vector of the dissemination of nosocomial infections. These results could reflect the epidemiological situation of the northwestern region of the country. Community surveillance and linkage to clinical samples in this hospital should be investigated. New surveillance strategies and strict hygiene rules measures are required in order to control the spread of resistant bacteria and to improve the healthcare system in our country. The intensification of hygiene measures is required to better control carbapenemases and ESBLs in Tunisia.

## Figures and Tables

**Table 1:** Number of Positive Bacterial sampled swabs and bacterial samples according to different wards, rooms and surfaces in the hospital.

Wards	Rooms	Positive Sampled Swabs Number *n*/N	Total
**Surgery**	Visceral 1	(13/13)	(48/104)
	Visceral 2	(10/25)	
	Gynecology	(8/17)	
	Orthopedic	(15/46)	
	Revealing Room	(2/2)	
	**Surface sites**	**bacterial samples Number**	**Total**
	Serum support	10	65
	Arm Blood Pressure Monitor	3	
	Door wrist	3	
	Oxygen mask	11	
	Saturometer	4	
	Patient examination table	2	
	Appliance table	4	
	Over bed Lifting Pole	2	
	Material Table	3	
	Tensiometer	2	
	Oxygen source	4	
	Serums Table	3	
	Cart	5	
	Light socket	1	
	Floor	2	
	Hardware tables intubation	2	
	Surgical suction source	1	
	Drape	3	
**Intensive Care Unit**	**Rooms**	**Positive Sampled Swabs Number *n*/N**	**Total**
	Reanimation 1	(11/24)	(20/41)
	Reanimation 2	(8/12)	
	Reanimation 3	(1/5)	
	**Surface sites**	**bacterial samples Number**	**Total**
	Mattress	6	48
	Oxygen embu	9	
	Serum support	6	
	Door	4	
	Door wrist	2	
	Oxygen mask	7	
	Material Tray	11	
	Arm Blood Pressure Monitor	3	
**Gastroenterology**	**Rooms**	**Positive Sampled Swabs Number n/N**	**Total**
	Rectoscopy	(5/9)	(10/17)
	Fibroscopy	(5/8)	
	**Surface sites**	**bacterial samples Number**	**Total**
	Fiberscope tube	9	39
	Rectoscope	4	
	Anoscope	4	
	Drape	4	
	Colonoscope hose	4	
	Colonoscope forceps	5	
	Cannula	7	
	Fiberscope forceps	2	

**Table 2:** Susceptibility of fermentative GNB against tested antibiotics.

Species	Number	Phenotype	Antibiogram															
			AMX	AMC	TZP	KF	CRO	PEF	ETP	IPM	CS	AK	CN	CIP	FF	F NIT	DO	SXT
***E. coli***	27	R	92.6	85.2	40.7	77.8	40.7	74.1	37	37	11.1	37	63	77.8	25.9	18.5	33.3	63.0
		I	0	0	7.40	14.8	0	7.40	0	0	0	0	0	0	0	0	0	0
		S	7.40	14.8	51.9	7.40	59.3	25.9	63	63	88.9	63	37	22.2	74.1	81.5	66.7	37.0
***E. cloacae***	46	R	100	100	0	100	13	39.1	4.3	2.1	6.5	0	8.7	63	39.1	65.2	2.2	4.3
		I	0	0	0	0	0	0	0	0	0	2.2	2.17	0	0	0	0	0
		S	0	0	100	0	87	60.9	95.7	97.9	93.5	97.8	89.1	37	60.9	34.8	97.8	95.7
***E. aerogenes***	4	R	100	100	0	75	0	0	0	0	0	0	0	25	27	0	0	0
		I	0	0	0	0	0	0	0	0	0	0	0	0	0	0	0	0
		S	0	0	100	25	100	100	100	100	100	100	100	75	75	100	100	100
***E. cancerogenus***	4	R	100	0	50	0	25	50	50	25	25	25	25	75	75	0	0	100
		I	0	0	0	0	0	0	0	0	0	0	0	0	0	0	0	0
		S	0	100	50	100	75	50	50	75	75	75	75	25	25	100	100	0

(AMX: Amoxicillin, AMC: Amoxicillin-Clavulanic Acid, TZP: Piperacillin + tazobactam, KF: Cephalothin, CRO: Ceftriaxone, PEF: Pefloxacin, ETP: Ertapenem, IPM: Imipenem, CS: Colistin, AK: Amikacin, CN: Gentamicin, CIP: Ciprofloxacin, FF: Fosfomycin, F. NIT: Nitrofurantoin, DO: Doxycycline, SXT: Trimethoprim-Sulfamid. (Bio-Rad, Marnes-la-Coquette, France)).

**Table 3:** Susceptibility of non-fermentative GNB against tested antibiotics.

Isolates Species	Number	Resistance	Antibiogram															
			TIC	TIM	TZP	CAZ	PEFFEP	IPM	CS	AK	TOB	CIP	CN	FF	F	DO	SXT	RA
***P. aeruginosa***	54	R	25.9	33.3	0	0	11.1	5.6	0	5.6	3.70	3.70	5.6	100	100	100	100	100
		I	0	0	0	0	0	0	0	0	0	0	0	0	0	0	0	0
		S	74.1	66.7	100	100	88.9	94.4	100	94.4	96.3	96.3	94.4	0	0	0	0	0
***P. stutzeri***	8	R	0	1.75	0	0	0	0	1.75	0	0	0	0	100	100	100	50	25
		I	0	0	0	0	0	0	0	0	0	0	0	0	0	0	0	25
		S	100	87.5	100	100	100	100	87.5	100	100	100	100	0	0	0	50	50
***P. koreensis***	7	R	100	100	0	71.4	28.6	0	0	28.6	0	0	14.8	42.8	100	100	71.4	0
		I	0	0	0	0	0	0	0	0	0	0	0	0	0	0	0	57.1
		S	0	0	100	28.6	71.4	100	100	71.4	100	100	85.2	57.2	0	0	28.6	42.9
***S. maltophilia***	2	R	50	0	0	50	0	50	50	0	100	100	0	0	100	0	0	50
		I	0	0	0	0	50	0	0	0	0	0	0	0	0	0	0	0
		S	50	100	100	50	50	50	50	100	0	0	100	100	0	100	100	50

(TIC: Ticarcillin, TIM: Ticarcillin + Clavulanic Acid, TZP: Piperacillin + tazobactam, CAZ: Ceftazidime, PEF: Pefloxacin, IPM: Imipenem, CS: Colistin, AK: Amikacin, TOB: Tobramycin, CIP: Ciprofloxacin, CN: Gentamicin, FF: Fosfomycin, F. NIT: Nitrofurantoin, DO: Doxycycline, SXT: Trimethoprim-Sulfamid, RA: Rifampicin. (Bio-Rad, Marnes-la-Coquette, France)).

**Table 4:** Phenotypic and molecular characterization of MBL and ESBL producers isolated from hospital environment.

Isolates	Species	Wards	Rooms	Surface Sites	IsolationDate	Phenotypic Resistance Patterns	Carbapenemases and β-Lactamases Detected
28H	*E. coli*	ICU	REA 1	Oxygen mask	27 March 2017	AMX; AMC; TZP; KF; CRO; PEF; ETP; IPM; AK; CN; CIP; FF; SXT.	NDM-1; CTX-M-90; TEM-164
30H	*E. coli*	ICU	REA 2	Serum support	27 March 2017	AMX; AMC; TZP; KF; CRO; PEF; ETP; IPM; AK; CN; CIP; SXT.	NDM-1; CTX-M-69; TEM-198
40H	*E. coli*	ICU	REA 2	Oxygen mask	9 March 2017	AMX; AMC; TZP; KF; CRO; PEF; ETP; IPM; AK; CN; CIP; FF; SXT.	NDM-1; CTX-M-90; TEM-104
41H	*E. coli*	gastroenterology	RECTO	Colonoscope hose	11 April 2017	AMX; AMC; TZP; KF; CRO; PEF; ETP; IPM; AK; CN; CIP; FF; SXT.	NDM-1; CTX-M-69; TEM-164
100H	*E. coli*	gastroenterology	RECTO	Colonoscope forceps	11 April 2017	AMX; AMC; TZP; KF; CRO; PEF; ETP; IPM; AK; CN; CIP; FF; SXT.	NDM-1; CTX-M-90; TEM-104
120H	*E. coli*	gastroenterology	RECTO	Colonoscope hose	11 April 2017	AMX; AMC; TZP; KF; CRO; PEF; ETP; IPM; AK; CN; CIP; FF; SXT.	NDM-1; CTX-M-90; TEM-163
125H	*E. coli*	gastroenterology	FIBRO	Cannula	11 April 2017	AMX; AMC; TZP; KF; CRO; PEF; ETP; IPM; AK; CN; CIP; FF; SXT.	NDM-1; CTX-M-90; TEM-104
126H	*E. coli*	Surgery	GYNECO	Arm Blood Pressure Monitor	9 March 2017	AMX; AMC; TZPTPZ; KF; CRO; PEF; ETP; IPM; AK; CN; CIP; FF; SXT.	NDM-1; TEM-163
155H	*E. coli*	Surgery	GYNECO	Material Table	2 December 2017	AMX; AMC; TZP; KF; CRO; PEF; ETP; IPM; AK; CN; CIP; FF; SXT.	NDM-1; VIM-2; CTX-M-90; TEM-198
88H	*P. aeruginosa*	ICU	REA 2	Serum support	27 March 2017	TIC; IPM; CN; FF; F; DO; SXT; RA.	*OprD* mutation
96H	*P. aeruginosa*	ICU	REA 2	Serums Table	22 November 2017	TIC; IPM; FF; F; DO; SXT; RA.	*OprD* mutation
97H	*P. aeruginosa*	ICU	REA 2	Oxygen Embu	9 March 2017	TIC; IPM; FF; F; DO; SXT; RA.	*OprD* mutation; OXA-494;aph3-iib; catb7; TEM-116
6H	*E. coli*	ICU	REA 1	Mattress	27 March 2017	AMX; AMC; KF; CRO; PEF; CN; CIP; FF; F. NIT.	CTX-M-88; TEM-198
11H	*E. coli*	gastroenterology	FIBRO	Fiber optic hose	11 April 2017	AMX; KF; CRO; PEF; CN; CIP.	CTX-M-139; TEM-163
13H	*E. coli*	Surgery	ORTHOP	Door wrist	9 March 2017	AMX; AMC; KF; CRO; PEF; CN; CIP; FF; F. NIT.	CTX-M-88; TEM-163
98H	*E. coli*	ICU	REA 1	Material Tray	27 March 2017	AMX.	TEM-198
21H	*E. coli*	gastroenterology	RECTO	Anoscope	11 April 2017	AMX; AMC; KF; CIP.	TEM-198
2E4	*E. coli*	ICU	REA 1	Oxygen mask	27 March 2017	AMX; KF; CRO; AK; CN; CIP; FF.	TEM-163
4E5	*E. coli*	gastroenterology	RECTO	Colonoscope forceps	11 April 2017	AMX; AMC; TZP; KF; CRO.	TEM-198
17H	*E. cloacae*	ICU	REA 1	Mattress	27 March 2017	AMX; AMC; KF; CIP; FF; F. NIT.	CTX-M-88; TEM-198
4H	*E. cloacae*	ICU	REA 1	Oxygen embu	27 March 2017	AMX; AMC; KF; CRO; PEF; CN; CIP; F. NIT.	CTX-M-139; TEM-163
19H	*E. cloacae*	ICU	REA 1	Serum support	27 March 2017	AMX; AMC; KF; CRO; PEF; CN; CIP; F. NIT.	CTX-M-139; TEM-163
35H	*E. cloacae*	ICU	REA 1	Serum support	27 March 2017	AMX; AMC; KF; CRO; PEF; CIP; DO; SXT.	CTX-M-139
7H	*E. cloacae*	ICU	REA 1	Oxygen embu	27 March 2017	AMX; AMC; KF; CRO; PEF; CN; CIP.	CTX-M-139; TEM-163
17E5B	*P. aeruginosa*	gastroenterology	FIBRO	Fiber optic hose	11 April 2017	TIM; CN; FF; F; DO; SXT; RA.	TEM-1
81H	*P. stutzeri*	Surgery	REA 3	Serum support	11 April 2017	TIM; FF; F; DO; SXT; RA.	TEM-1
